# Can concentrations of steroid hormones in brown bear hair reveal age class?

**DOI:** 10.1093/conphys/coy001

**Published:** 2018-01-29

**Authors:** Marc Cattet, Gordon B Stenhouse, John Boulanger, David M Janz, Luciene Kapronczai, Jon E Swenson, Andreas Zedrosser

**Affiliations:** 1 RGL Recovery Wildlife Health & Veterinary Services, 415 Mount Allison Crescent, Saskatoon, Saskatchewan S7H 4A6, Canada; 2 Department of Veterinary Pathology, Western College of Veterinary Medicine, University of Saskatchewan, 52 Campus Drive, Saskatoon, Saskatchewan S7N 5B4, Canada; 3 fRI Research and Alberta Environment and Parks, 1176 Switzer Drive, Hinton, Alberta T7V 1X6, Canada; 4 Integrated Ecological Research, 924 Innes Street, Nelson, British Columbia V1L 5T2, Canada; 5 Department of Veterinary Biomedical Sciences, Western College of Veterinary Medicine, University of Saskatchewan, 52 Campus Drive, Saskatoon, Saskatchewan S7N 5B4, Canada; 6 Toxicology Centre, University of Saskatchewan, 44 Campus Drive, Saskatoon, Saskatchewan S7N 5B3, Canada; 7 Faculty of Environmental Sciences and Nature Resource Management, Norwegian University of Life Sciences, PO Box 5003, NO-1432 Ås, Norway and Norwegian Institute for Nature Research, Høgskoleringen 9, 7034 Trondheim, Norway; 8 Department of Natural Sciences and Environmental Health, Telemark University College of Southeast Norway, NO-3800 Bø i Telemark, Norway; 9 Department for Integrative Biology, Institute for Wildlife Biology and Game Management, University for Natural Resources and Life Sciences, Vienna A-1180, Austria

**Keywords:** Age class discrimination, brown bear, enzyme-linked immunosorbent assay (ELISA), hair steroid hormone profile, non-invasive hair collection, *Ursus arctos*

## Abstract

Although combining genetic and endocrine data from non-invasively collected hair samples has potential to improve the conservation of threatened mammals, few studies have evaluated this opportunity. In this study, we determined if steroid hormone (testosterone, progesterone, estradiol and cortisol) concentration profiles in 169 hair samples collected from free-ranging brown bears (*Ursus arctos*) could be used to accurately discriminate between immature and adult bears within each sex. Because hair samples were acquired opportunistically, we also needed to establish if interactions between hormones and several non-hormone factors (ordinal day, year, contact method, study area) were associated with age class. For each sex, we first compared a suite of candidate models by Akaike Information Criteria model selection, using different adult-age thresholds (3, 4 and 5 years), to determine the most supported adult age. Because hair hormone levels better reflect the endocrine state at an earlier time, possibly during the previous year, then at the time of sampling, we re-analysed the data, excluding the records for bears at the adult-age threshold, to establish if classification accuracy improved. For both sexes, candidate models were most supported based on a 3-year-old adult-age threshold. Classification accuracy did not improve with the 3-year-old bear data excluded. Male age class was predicted with a high degree of accuracy (88.4%) based on the concomitant concentrations of all four hormones. Female age class was predicted with less accuracy (77.1%) based only on testosterone and cortisol. Accuracy was reduced for females, primarily because we had poor success in correctly classifying immature bears (60%) whereas classification success for adult females was similar to that for males (84.5%). Given the small and unbalanced sample used in this study, our findings should be viewed as preliminary, but they should also provide a basis for more comprehensive future studies.

## Teaser

We evaluated if steroid hormone concentration profiles in hair collected from brown bears could be used to discriminate between immature and adult bears. Classification accuracy was excellent for male age classes. We also had good success with accurately classifying adult females, but poor accuracy with classifying immature females.

## Introduction

Hair is one of several biological media that can be collected non-invasively from wild mammals. It is relatively easy and inexpensive to collect or ‘trap’ (Woods *et al.*, 1999; Patkó *et al.*, 2016). In addition, hair is easy and inexpensive to store for laboratory analysis; it is simply air-dried, sealed in paper envelopes, with or without a dessicant, and stored in a cool dry location (Long *et al.*, 2007; Kendall and McKelvey, 2008). Hair collected from wildlife has been analysed in various ways, and for various purposes, including identification of individuals based on macro- and microscopic features (Stains, 1958; de Marinis and Asprea, 2006), genetic sampling of populations based on DNA extraction from hair follicles (Woods *et al.*, 1999; Wilson *et al.*, 2016), endocrine system function of individuals based on steroid hormone levels in hair (Koren *et al.*, 2002; Schell *et al.*, 2017), migration patterns and/or dietary habits of individuals based on stable isotope levels (Hobson, 1999; Cerling *et al.*, 2006), and contaminant exposure of individuals based on hair levels of toxicants (d’Havé *et al.*, 2005; Hernout *et al.*, 2016). These research pursuits have generally followed independent paths, with combined studies being relatively uncommon, although several recent studies have combined genetic, endocrine and/or stable isotope analyses of hair to address issues relevant to methodology (Sergiel *et al.*, 2017) and ecology (Bryan *et al.*, 2013; Lafferty *et al.*, 2015). This contrasts with numerous studies based on non-invasive faecal collections in which DNA and hormone metabolites have been measured in tandem to increase understanding of the state of free-ranging populations (Wasser *et al.*, 2004; Mesa-Cruz *et al.* 2016; Rehnus and Palme, 2017)

For the past 10 years, we have been evaluating the potential for hair hormone analyses to support the conservation of brown bears, with a primary focus on populations in Alberta, Canada (Macbeth *et al.*, 2010; Bourbonnais *et al.*, 2013, 2014; Cattet *et al.*, 2014, 2017; Kroshko *et al.*, 2017). Brown bears in Alberta are considered a ‘threatened species’ based on current estimates of population size (ASRD and ACA, 2010) combined with unsustainable levels of human-caused mortality (Benn and Herrero, 2002; Nielsen *et al.*, 2004). This situation has been further exacerbated by the individual- and population-level effects of increasing levels of human use within brown bear habitat (Boulanger *et al.*, 2013; Nielsen *et al.*, 2013; Boulanger and Stenhouse, 2014). Among large carnivores, brown bears are known to have low resilience (ability for an individual to experience disturbance and maintain normal physiological processes) (Martin and Wiebe, 2004) in the face of ongoing human-caused landscape disturbance (Weaver *et al.*, 1996). Key conservation actions have included a province-wide hunting moratorium that was instituted in 2006, a species recovery plan that was established in 2008, designation of the species as threatened in 2010, and a revised recovery plan that was released in draft form for public input in June 2016 (ASRD, 2008; ASRD and ACA, 2010; AEP, 2016). In addition, DNA-based capture-mark-recapture studies were conducted from 2004 to 2008 with a different population inventoried in each of these years. One of these populations was re-assessed in the same manner in 2014 and, from this, it appears likely that non-invasive genetic hair sampling methods will continue as the basis for estimating and monitoring sizes of provincial populations (Stenhouse *et al.*, 2015). With this realization, we are moving in our research toward augmenting population characteristics derived from these studies (population size, density, spatial distribution, sex ratio) with health information obtained from the hairs snagged by barbed wire from individual bears.

Our research into the utility of hair hormone analyses for brown bear conservation has been greatly strengthened through collaboration with the Scandinavian Brown Bear Research Project, which has provided a comparative basis to try to understand the response of brown bears to human-caused landscape disturbance, and to more effectively conserve their populations in Alberta. In Scandinavia, brown bears were almost wiped out in at the end of the 19th century through predator extermination programmes (Swenson *et al.*, 1995). By the 1930s, roughly 130 individuals were estimated to remain in Sweden, whereas brown bears in Norway were even closer to extinction (Swenson *et al.*, 1994, 1995). However, actions to conserve brown bears that began in the late 1800s in Sweden and later in Norway have proven successful to their recovery. The 1984 launch of a long-term research programme, the Scandinavian Brown Bear Research Project, to provide management authorities with science-based information to help them reach their management and conservation goals was a significant step in this effort. Today, the brown bear population in Scandinavia (Sweden and Norway) is believed to be stable at around 3000 individuals (Aarnes *et al.*, 2014; Kindberg and Swenson, 2014; but see Swenson *et al.*, 2017). Meanwhile, the research programme has contributed a wealth of general knowledge about brown bear ecology as well as specific knowledge about population dynamics, the mechanisms of population expansion, conservation genetics and the effects of hunting (which was introduced in 1943) on Scandinavian brown bears.

This study closely follows a study in which we developed laboratory procedures to quantify reproductive steroid hormone (testosterone, progesterone, and estradiol) concentrations in brown bear hair and then verified that changes in the concentrations of these hormones in hair collected from captive adult brown bears corresponded with key reproductive events, including breeding and pregnancy (Cattet *et al.*, 2017). In the current study, we evaluate if steroid hormone (testosterone, progesterone, estradiol and cortisol) concentration profiles in hair samples collected from free-ranging brown bears could be used to effectively discriminate between immature and adult bears within each sex. In the context of non-invasive genetic hair sampling, discrimination between sexes on the basis of hormone profiles is not necessary, because sex would be confirmed through DNA analysis. At this time, however, DNA analysis does not enable the assignment of age or age class. Nonetheless, knowledge of age structure is essential to the success of many wildlife management and conservation programmes (Doak and Cutler, 2014; Woodruff *et al.*, 2016). For example, in reference to Alberta’s efforts for brown bear recovery, it would be invaluable to be able to determine from non-invasive genetic hair sampling if adult females, who will presumably breed, are occupying priority areas identified for brown bear conservation. To our knowledge, the effect of puberty on steroid hormone concentrations has not been investigated in brown bears. However, consistent changes in steroid hormone levels associated with sexual maturation have been identified in different biological media in other species, including in the blood plasma of spotted hyaenas (*Crocuta crocuta*; Glickman *et al.*, 1992), in the faeces of giant pandas (*Ailuropoda melanoleuca*; Kersey *et al.*, 2010), and in the hair of several non-human primate species (Fourie *et al.*, 2016).

## Methods

### Sources of brown bear hair and aging of bears

We opportunistically obtained 169 hair samples (~150 g per sample) collected under the two long-term research projects described above (Tables S1 and S2, Figs S1 and S2—Note that table or figure numbers preceded by ‘S’ herein refer to results presented in the Supplementary Information). In total, 36 samples were collected in Alberta by shaving hair at the skin surface from the shoulder of free-ranging brown bears (19 females from 1.4 to 11.7 years, 17 males from 2.4 to 19.4 years) from June 2009 to September 2014. Of these, 15 samples were collected from bears captured by remote drug delivery from helicopter and 21 from bears captured by culvert trap. Details concerning capture and handling procedures used in Alberta are provided in Cattet *et al.* (2008). Captures were approved by the University of Saskatchewan’s Committee on Animal Care and Supply (Animal Use Protocol # 20 010 016) and were in accordance with guidelines provided by the Canadian Council on Animal Care for the safe handling of wildlife (CCAC, 2003) and the American Society of Mammalogists’ Animal Care and Use Committee (Sikes and Gannon, 2011).

Overall, 133 hair samples were collected in Sweden by pulling (plucking) hair from the skin of brown bears (64 females from 1.3 to 22.3 years, 69 males from 1.3 to 22.7 years). Of these, 63 were captured by remote drug delivery from helicopter from April 2000 to April 2007 and 70 were legally killed by hunters from August to October 2008. Samples from captured bears were consistently plucked from the shoulder and examiners were instructed to pluck samples from hunter-killed bears from the shoulder. The 63 captured bears were composed of 20 adult females and their dependent offspring that were all 1.3 years old (i.e. yearlings) with litters ranging in size from 1 to 3 offspring. Details concerning capture and handling procedures used in Sweden are provided in Arnemo and Evans (2017). All captures were approved by the Swedish Ethical Committee on Animal Research (application numbers C 7/12 and C 18/15) and the Swedish Environmental Protection Agency.

Although hair collection methods varied between projects (i.e. shaving in Alberta vs plucking in Sweden), hair samples were handled in a similar manner in that they never came in direct contact with human skin, and were placed into a paper envelope either using forceps or by hand while wearing examination gloves. The envelopes were left open for several hours to ensure that samples were air-dried, and then sealed and stored under low light at room temperature in Alberta, and at −18°C in Sweden, until the analysis of hormone levels in the following 6 months to 14 years.

Because age class was a variable of primary interest in this study, it should be noted that in both Alberta, Canada and Sweden, the ages of all bears of uncertain age were estimated by counting the cementum annuli of an extracted premolar (Stoneberg and Jonkel, 1966; Matson *et al.*, 1993).

### Sample preparation, hormone extraction and analyses

Only guard hairs were analysed and, in cases where samples had been plucked, follicles were removed with sharp scissors prior to decontamination and hormone extraction. We removed gross contaminants (e.g. mud, dried faeces) with fine forceps and gentle agitation of hairs, while taking care not to damage hair shafts. Approximately 125 mg of hair was prepared as a single batch for extraction of all four hormones to ensure each sub-sample (one for each hormone) was handled identically. Hair samples were washed to remove external contamination and ground to powder as described in detail by Cattet *et al.* (2017). Powdered hair was collected and transferred to a 1.5 ml tube and stored in the dark at room temperature prior to hormone extraction.

For powdered hair, we used the protocol developed and validated for brown bear hair by Macbeth *et al.* (2010) to extract cortisol, which was subsequently validated in brown bear hair for progesterone and testosterone (Cattet *et al.*, 2017). HPLC grade methanol (1.5 ml) was added to 75 mg of ground hair (0.02 ml methanol per mg of powdered hair; 25 mg for each hormone) and blended with a vortex mixer for 10 s. The mixture was placed in a slow end-over-end rotator at room temperature for 24 h, after which it was centrifuged at 4500 rpm for 15 min at 20°C. Equal volumes of supernatant were collected into 12 × 75 mm^2^ glass culture tubes, and the solvent was evaporated under a gentle stream of nitrogen gas. The extracted powder was re-extracted twice with another 1.5 ml of methanol each time, blended with a vortex mixer for 40 s, centrifuged and the supernatant was collected as before. The final combined extract was divided into three identical volumes in separate tubes and concentrated to the bottoms of the tubes, using consecutive rinses of methanol in decreasing volumes (0.4, 0.2, 0.15 ml), and dried under nitrogen gas after each rinse.

For estradiol extraction, we used a protocol previously validated for brown bear hair (Cattet *et al.*, 2017). Briefly, we added 10 ml of methyl tert-butyl ether (EMD Chemicals, Gibbstown, NJ) to 50 mg of powdered hair sample in a 16 × 125 mm^2^ glass culture tube, blended it with a vortex mixer for 10 s, and placed the mixture in a slow rotator for 24 h. Immediately afterward, we centrifuged the mixture at 4500 rpm for 15 min at 20°C, collected the supernatant, and dried it under nitrogen gas. Similar to the extraction described above for cortisol, testosterone and progesterone, the extracted powder was re-extracted an additional two times with 10 ml of methyl tert-butyl ether, and resulting combined extracts were concentrated to the bottoms of tubes.

The extracted hormones were reconstituted in 125–250 μl of buffers provided by the respective enzyme-linked immunosorbent assay (ELISA) kits (cortisol—Oxford Biomedical, pre-2016 EA65 ELISA kit, Rochester Hills, MI, USA; progesterone—Enzo Life Sciences, ADI-900-011 ELISA kit, Ann Arbor, MI, USA; testosterone—Enzo Life Sciences, ADI-900-065; estradiol—Calbiotech, ES180S ELISA kit, Spring Valley, CA, USA). Hormone extracts were reconstituted in the minimal volume of buffer that would allow us to maximally concentrate hormones and run samples in duplicate on the respective ELISAs (250 μl for testosterone and progesterone, 200 μl for cortisol, and 125 μl for estradiol). We selected the various kits based on their sensitivity (i.e. lowest standard) and the volume of sample required to run in duplicate (due to the small volumes of reconstituted hormone extracts obtained). We reconstituted all samples for 12 h in the dark at 4°C. Samples were then gently mixed before centrifugation at 4500 rpm for 5 min at 20°C. We diluted samples with the appropriate kit buffer in order for the ELISA result to fall on the linear portion of the standard curve, based on preliminary analyses conducted during assay validation (progesterone 1/10, testosterone 1/5, cortisol and estradiol undiluted) with the appropriate buffer provided with each kit. The performance characteristics of each ELISA (lower limits of detection, parallelism and extraction efficiency) are described in detail in Cattet *et al.* (2017). Intra- and inter-assay percent coefficients of variation (%CV; SD/mean × 100%) for each assay were <10 and <15%, respectively (Cattet *et al.*, 2017). In the case of a sample having a %CV >15% in an assay, or falling outside the linear portion of the standard curve, that sample was re-run on the ELISA until this value was acceptable. A standard curve was run on every plate. All samples were analysed over a 6-month period in 2014–15, with the sequence of samples selected in a randomized manner.

### Statistical analysis

We performed all statistical analyses using R 3.3.2 (R Core Team, 2016). Each of the 169 records in this study represented a unique individual, i.e. no repeated measures (Tables S1-S2). We adapted the protocol for data exploration described in Zuur *et al.* (2010), using Cleveland dot-plots to evaluate continuous and discrete variables for potential outliers, and pair-plots (Pearson *r* ≥ 0.70) and generalized variance inflation factors (GVIF^1/(2·df)^ ≥ 3.0) to identify collinear variables (Table 1). This was facilitated by using the ‘lattice’ package (Sarkar, 2008) in R, as well as the custom R code provided by Ieno and Zuur (2015).

**Table 1:** Variables evaluated as potential determinants of the age class (immature or adult) of 86 male brown bears and 83 female brown bears that were either captured in Alberta, Canada (*N* = 36) or Sweden (*N* = 63), or killed legally Sweden (*N* = 70), between 2000 and 2014

Attribute	Predictor variable (abbreviation)	Variable type	Values
Hormone	Testosterone (test)	Continuous	0.2–27.6 pg/mg
	Progesterone (prog)	Continuous	0.3–17.4 pg/mg
	Estradiol (est)	Continuous	0.0043–0.0261 pg/mg
	Cortisol (cort)	Continuous	0.33–12.99 pg/mg
Time	Adjusted ordinal day^a^ (d)	Discrete	1–365 with March 21 set as Day 1
	Year (y)	Discrete	2000–14
Contact method	Contact method (cm)	Categorical	culvert trap capture, remote drug delivery from helicopter capture or legally killed
Study area	Study area (sa)	Categorical	Alberta or Sweden

^a^Adjusted ordinal day is the day on which a bear was captured or killed, and sampled. March 21 was set as Day 1 to represent the approximate time that a bear emerged from its den.

Following data exploration, we used the ‘glm’ function in package ‘stats’ (R Core Team, 2016) to analyse the male and female data with a binomial response that was either immature or adult. First, however, we standardized all continuous predictor variables by subtracting the mean from the observed values and dividing by the standard deviation. This was done to reduce multicollinearity and the associated problems that are caused by two-way interactions when calculating model coefficients. We then developed six candidate models to be used in ten separate analyses, five for each sex. The candidate models were composed of the following predictor variables, as shown in Table 2:

- Model 1—an intercept-only (null) model;
- Model 2—only hormones in linear (x) and polynomial forms (x^2^ and x^3^) without interactions;
- Model 3—only hormones with interactions;
- Model 4—hormones + (hormone × time) interactions;
- Model 5—hormones + (hormone × contact method) interactions; and
- Model 6—hormones + (hormone × study area) interactions.

**Table 2:** Candidate models evaluated to predict the age class (immature or adult) of 86 male brown bears and 83 female brown bears. For each model, with the exception of the null model, we refined an initial model that contained all variables of interest to a final model by backward elimination of variables. Each final candidate model was selected by comparing ΔAICc values, i.e. ΔAICc = 0.00

Model	Initial structure	Refining procedure
1. Null	Intercept only	None
2. Hormone^a^ only (no interactions)	test + test^2^ + test^3^ + prog + prog^2^ + prog^3^ + est + est^2^ + est^3^ + cort + cort^2^ + cort^3^	Backward elimination
3. Hormone only (with interactions)	Most supported model 2 + all two-way interactions	Backward elimination
4. Hormone + time	Most supported model 3 + all hormone × day interactions + all hormone × year interactions	Backward elimination
5. Hormone + contact method	Most supported model 3 + all hormone × contact method interactions	Backward elimination
6. Hormone + study area	Most supported model 3 + all hormone × study area interactions	Backward elimination

^a^Hormones are testosterone (test), progesterone (prog), estradiol (est) and cortisol (cort).

For Model 2, we began with a global model that incorporated all four hormones in their linear and polynomial forms, and then refined it by backward elimination using the ‘drop1’ function in package ‘lme4’ (Bates *et al.*, 2015) to successively remove the least significant variable, based on *P* values with a significance level of *α* = 0.05, and re-fit the reduced model. We compared among successively-refined models using Aikaike Information Criteria (AIC) model selection (Burnham and Anderson, 2002). For Model 3, we added all possible two-way interactions to the most supported structure (∆AIC_C_ = 0.00) for Model 2, and then refined it by backward elimination. For Models 4–6, we followed the same procedure, but used the most supported structure for Model 3 instead of the most supported structure for Model 2. We avoided using candidate models with combinations of year, contact method, and study area because of the small sample size and because we could not separate the effects of these particular predictor variables (i.e. confounded variables) (Tables S1 and S2, Figs S1 and S2).

Within each sex, we conducted three separate analyses, each using a different threshold age for adulthood – 3, 4 or 5 years. Within each analysis, we compared the six candidate models (Table 2) using AIC model selection (Burnham and Anderson, 2002) to determine the most supported model (∆AIC_C_ = 0.00) at each adult-age threshold. In a fourth analysis, we compared between the most supported models at each adult-age threshold to identify which threshold had the most support overall. In a fifth and final analysis, we excluded all records at the most supported adult-age threshold, and re-analysed the data using the same approach shown in Table 2. We predicted that the exclusion of data at the adult-age threshold would increase accuracy in discriminating between age classes if hormone levels in hair were more indicative of the bear’s endocrine state at a previous time (e.g. prior to hibernation when hair was growing) than at the time of sampling, e.g. during spring when hair growth was arrested.

We used several measures of classification accuracy to evaluate candidate models for their effectiveness in discriminating between age classes. The simplest measure was the number of correct predictions divided by the sample size, expressed as a percentage, and referred to as ‘Accuracy (%)’ in Tables 3a–6a. We also used the mean area under the curve (AUC), formally termed the area under the receiver operating characteristic (ROC) curve, which is a more complex measure. This index provides a single measure of overall classification accuracy between 0.5 and 1.0 that is not dependent upon a particular threshold (Deleo, 1993). In the context of this study, an AUC value of 0.5 would indicate that hair hormone profiles did not differ between age classes, whereas a score of 1.0 would indicate no overlap (perfect discrimination) in hormone profiles. We calculated the mean AUC and 95% confidence interval values using the ‘roc’ and ‘ci’ functions in package ‘pROC’ (Robin *et al.*, 2011). The other measures of classification accuracy that we used were sensitivity and specificity. In contrast to AUC values, these are threshold-dependent measures, i.e. values <0.5 = 0, and values ≥0.5 = 1. In the context of this study, sensitivity refers to the number of bears that were correctly classified as belonging to a particular class divided by the observed (true) number of bears within that class. Conversely, specificity refers to the number of bears that were correctly classified as not belonging to a particular class divided by the observed (true) number of bears outside of that class. We calculated sensitivity and specificity values (%) from confusion matrices for each model (Tables S5-S6) that were constructed using the ‘xtabs’ function in package ‘stats’ (R Core Team, 2016).

**Table 3a:** Candidate models that were most supported (∆AIC_C_ = 0.00) for predicting the age class (immature or adult) of 86 male brown bears. Each model was the outcome of one of four individual analyses^a^. For the first three analyses, we used different age thresholds (≥3, ≥4 or ≥5 years) for adulthood while retaining all records in the data set. For the final analysis, we re-analysed the data set that was used for the most supported model (∆AIC_C_ = 0.00), but excluded the records for bears at the threshold age (in this case, 3 years old)

Adult age (y)	*N*	Model	Most supported model structure^b^	*K*	AIC_C_	∆AIC_C_	*w*_*i*_	Accuracy (%)
≥3	86	Hormone + time	test + (cort × d) + cort^3^ + (prog × est^3^)	9	64.87	0.00	0.97	88.4
≥5	86	Hormone + contact method	(test × cm) + prog	7	65.33	0.46	0.87	83.7
≥4	86	Hormone + contact method	test + (cort × cm) + cort^2^ + (prog × cm)	11	71.89	7.02	0.41	86.1
Model based on reduced data set excluding records for 3-year-old bears								
≥3	80	Hormone + contact method	(test × cm) + (est^3^ × cm) + cort^2^	10	61.14	0.00	0.55	87.5

^a^Statistics are number of estimable parameters in model (*K*), sample-size-adjusted Akaike information criterion (AIC_C_), difference in AIC_C_ between top model and model *i* (∆AIC_C_), Akaike weight (*w*_*i*_) and accuracy (%).

^b^Variables are testosterone (test), progesterone (prog), estradiol (est), cortisol (cort), ordinal day (d) and contact method (cm).

**Table 3b:** Comparison of candidate models in Table 3a. by mean Area Under the Curve (AUC), sensitivity (Sen, %) and specificity (Spe, %) to predict the age class for 86 male brown bears

Adult age (y)	*N*	Immature			Adult		
		Mean AUC (95% CI)	Sen	Spe	Mean AUC (95% CI)	Sen	Spe
≥3	86	0.96 (0.92–0.99)	83.3	93.2	0.96 (0.92–0.99)	93.2	83.3
≥5	86	0.94 (0.89–0.98)	86.5	79.4	0.94 (0.89–0.98)	79.4	86.5
≥4	86	0.95 (0.91–0.99)	87.5	84.2	0.95 (0.91–0.99)	84.2	87.5
Model based on reduced data set excluding records for 3-year-old bears							
≥3	80	0.96 (0.93–0.99)	85.7	89.5	0.96 (0.93–0.99)	89.5	85.7

**Table 4a:** Candidate models that were most supported (∆AIC_C_ = 0.00) for predicting the age class (immature or adult) of 83 female brown bears. Each model was the outcome of one of four individual analyses^a^. For the first three analyses, we used different age thresholds (≥3, ≥4 or ≥5 years) for adulthood while retaining all records in the data set. For the final analysis, we re-analysed the data set that was used for the most supported model (∆AIC_C_ = 0.00), but excluded the records for bears at the threshold age (in this case, 3 year olds)

Adult age (y)	*N*	Model	Most supported model structure^b^	*K*	AIC_C_	∆AIC_C_	*w*_*i*_	Accuracy (%)
≥3	83	Hormone + study area	(test × cort^3^) + test^2^ + cort + (test × sa)	7	80.10	0.00	0.91	77.1
≥5	83	Hormone + study area	(cort × sa) + (test^2^ × sa) + (cort^3^ × sa)	8	86.84	6.74	0.99	74.7
≥4	83	Hormone + study area	(test^2^ × sa) + est^3^	5	93.28	13.18	0.90	77.1
Model based on reduced data set excluding records for 3-year-old bears								
≥3	77	Hormone only (with interactions)	test^2^ + prog^3^ + (prog × cort) + est + (prog × cort^3^)	9	84.97	0.00	0.49	79.2

^a^Statistics are number of estimable parameters in model (*K*), sample-size-adjusted Akaike information criterion (AIC_C_), difference in AIC_C_ between top model and model *i* (∆AIC_C_), Akaike weight (*w*_*i*_) and accuracy (%).

^b^Variables are testosterone (test), progesterone (prog), estradiol (est), cortisol (cort), ordinal day (d) and study area (sa).

**Table 4b:** Comparison of candidate models in Table 4a by mean area under the curve (AUC), sensitivity (Sen, %) and specificity (Spe, %) to predict the age class for 83 female brown bears

Adult age (y)	*N*	Immature			Adult		
		Mean AUC (95% CI)	Sen	Spe	Mean AUC (95% CI)	Sen	Spe
≥3	83	0.87 (0.79–0.94)	60.0	84.5	0.87 (0.79–0.94)	84.5	60.0
≥5	83	0.87 (0.80–0.94)	74.3	75.0	0.87 (0.80–0.94)	75.0	74.3
≥4	83	0.82 (0.73–0.92)	67.7	82.7	0.82 (0.73–0.92)	82.7	67.7
Model based on reduced data set excluding records for 3-year-old bears							
≥3	3-yr-olds (80)	0.87 (0.78–0.95)	60.0	88.5	0.87 (0.78–0.95)	88.5	60.0

**Table 5a:** Comparison^a^ of candidate models^b^ by Aikaike Information Criteria (AIC) model selection to predict the age class for 86 male brown bears. Males < 3 years old were classified as immature (*N* = 42) whereas males ≥3 years old were classified as adult (*N* = 44). Model M1 is an intercept-only (null) model

Model	Hormone	Time	Study area	Contact method	Interactions	*K*	AIC_C_	∆AIC_C_	*w*_*i*_	Accuracy (%)
M4	test, prog, est^3^, cort, cort^3^	d			(prog × est^3^), (cort × d)	9	64.87	0.00	0.97	88.4
M6	test, prog, est^3^ cort, cort^3^		sa		(prog × est^3^), (cort × sa), (cort^3^ × sa)	10	73.59	8.72	0.01	86.1
M5	test, est^3^			cm	(est^3^ × cm)	7	74.39	9.52	0.01	84.9
M3	test, prog, est^3^, cort, cort^3^				(test × est^3^), (prog × est^3^)	8	74.44	9.57	0.01	84.9
M2	test, prog, est^3^, cort, cort^3^					6	80.29	15.42	0.00	86.1
M1						1	121.22	56.35	0.00	50.0

^a^Statistics are number of estimable parameters in model (*K*), sample-size-adjusted Akaike information criterion (AIC_C_), difference in AIC_C_ between top model and model *i* (∆AIC_C_), Akaike weight for model *i* (*w*_*i*_) and accuracy (%).

^b^Variables are testosterone (test), progesterone (prog), estradiol (est), cortisol (cort), ordinal day (d), study area (sa) and contact method (cm).

**Table 5b:** Comparison of candidate models in Table 5a. by mean Area Under the Curve (AUC), sensitivity (Sen, %) and specificity (Spe, %) to predict the age class for 86 male brown bears

Model^a^	Immature (*N* = 42)			Adult (*N* = 44)		
	Mean AUC (95% CI)	Sen	Spe	Mean AUC (95% CI)	Sen	Spe
M4	0.96 (0.92–0.99)	83.3	93.2	0.96 (0.92–0.99)	93.2	83.3
M6	0.95 (0.90–0.99)	85.7	86.4	0.95 (0.90–0.99)	86.4	85.7
M5	0.93 (0.87–0.98)	78.6	90.0	0.93 (0.87–0.98)	90.9	78.6
M3	0.93 (0.88–0.98)	83.3	86.4	0.93 (0.88–0.98)	86.4	83.3
M2	0.90 (0.84–0.97)	85.7	86.4	0.90 (0.84–0.97)	86.4	85.7

^a^Model M1 is excluded from this table because it is an intercept-only (null) model.

**Table 6a:** Comparison^a^ of candidate models^b^ by Aikaike Information Criteria (AIC) model selection to predict the age class and presence of offspring for 83 female brown bears. Females < 3 years old were classified as immature (*N* = 25) whereas females ≥3 years old, which included solitary females (*N* = 33) and all females with offspring (*N* = 25), were classified as adult. Model F1 is an intercept-only (null) model

Model	Hormone	Time	Study area	Contact method	Interactions	*K*	AIC_C_	∆AIC_C_	*w*_*i*_	Accuracy (%)
F6	test, test^2^, cort, cort^3^		sa		(test × sa), (test × cort^3^)	7	80.10	0.00	0.91	77.1
F4	test, test^2^, prog^3^, cort, cort^3^	y			(test × prog^3^), (cort × y)	9	86.99	6.89	0.03	80.7
F3	test, test^2^, prog^3^, est, cort, cort^3^				(test × prog^3^)	8	87.06	6.95	0.03	78.3
F2	test^2^, prog, prog^3^, est, cort, cort^3^					7	87.51	7.41	0.02	77.1
F5	test, test^2^, prog^3^, est, cort, cort^3^			cm	(test × prog^3^), (est × cm)	10	88.28	8.18	0.02	77.1
F1						1	103.62	23.52	0.00	50.0

^a^Statistics are number of estimable parameters in model (*K*), sample-size-adjusted Akaike information criterion (AIC_C_), difference in AIC_C_ between top model and model *i* (∆AIC_C_), Akaike weight for model *i* (*w*_*i*_), and accuracy (%).

^b^Variables are testosterone (test), progesterone (prog), estradiol (est), cortisol (cort), year (y), study area (sa) and contact method (cm).

**Table 6b:** Comparison of candidate models in Table 6a. by mean area under the curve (AUC), sensitivity (Sen, %) and specificity (Spe, %) to predict the age class for 83 female brown bears

	Immature (*N* = 25)			Adult (*N* = 58)		
Model^a^	Mean AUC (95% CI)	Sen	Spe	Mean AUC (95% CI)	Sen	Spe
F6	0.87 (0.79–0.93)	60.0	84.5	0.87 (0.79–0.93)	84.5	60.0
F4	0.87 (0.79–0.94)	68.0	86.2	0.87 (0.79–0.94)	86.2	68.0
F3	0.85 (0.77–0.93)	56.0	87.9	0.85 (0.77–0.93)	87.9	56.0
F2	0.84 (0.76–0.93)	48.0	89.7	0.84 (0.76–0.93)	89.7	48.0
F5	0.86 (0.79–0.94)	56.0	86.2	0.86 (0.79–0.94)	86.2	56.0

^a^Model F1 is excluded from this table because it is an intercept-only (null) model.

For the most supported models (∆AIC_C_ = 0.00) for each sex, we also constructed ROC curves (Swets, 1979) for each age class using the ‘plot.roc’ function in package ‘pROC’ (Robin *et al.*, 2011). ROC curves illustrate the trade-off between sensitivity and specificity (graphically presented as 1—specificity) over the range of all possible thresholds (cut-off points).

## Results

Through the exploratory data analysis, we did not detect any outlying values within the hormone and time variables. Thus, we proceeded to use all records, variables and values in subsequent analyses. As expected though, we found that contact method and country were collinear (GVIF^1/(2·df)^ ≥ 3.0) with each other, and with year and ordinal day, and therefore could not be used in combination in the candidate models (Table 2). However, year and ordinal day were not collinear (GVIF^1/(2·df)^ < 3.0) and, therefore, were used together in the same candidate model (i.e. Model 4 in Table 2).

**Figure 1:**
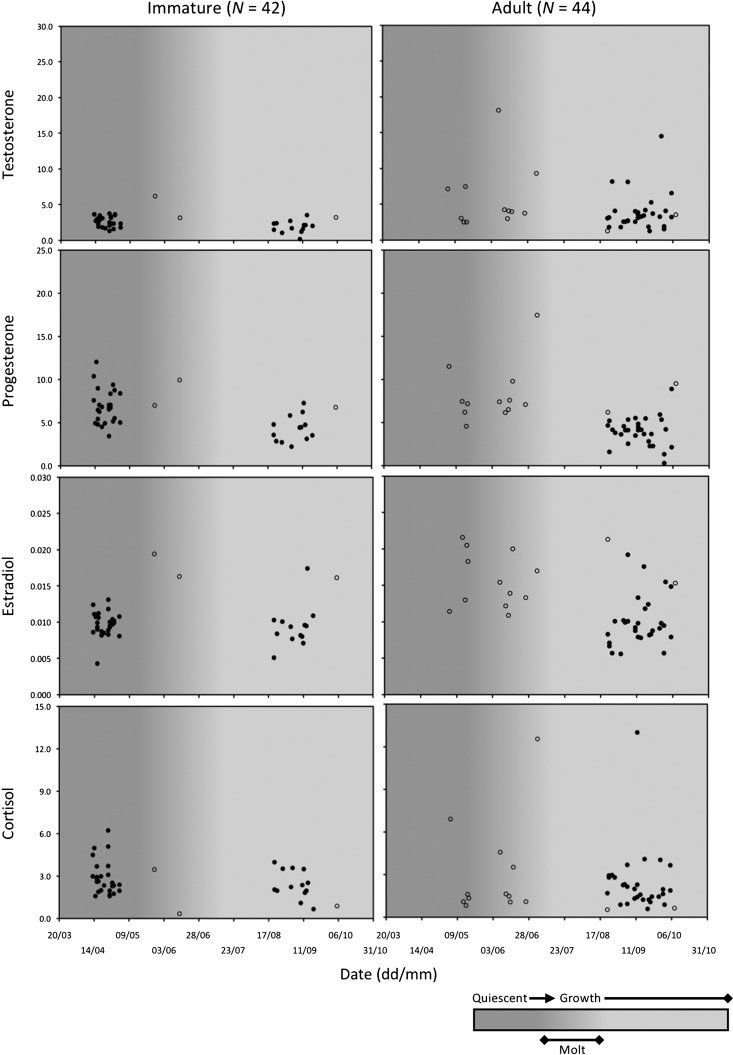
Hair hormone levels in 86 male brown bears in relation to time of year and phase of hair growth cycle (quiescent, molt, growth). Open circles represent bears captured in Alberta, Canada (*N* = 17). Closed circles represent bears captured or killed in Sweden (*N* = 69). Males < 3 years old were classified as immature whereas males ≥3 years old were classified as adult.

**Figure 2:**
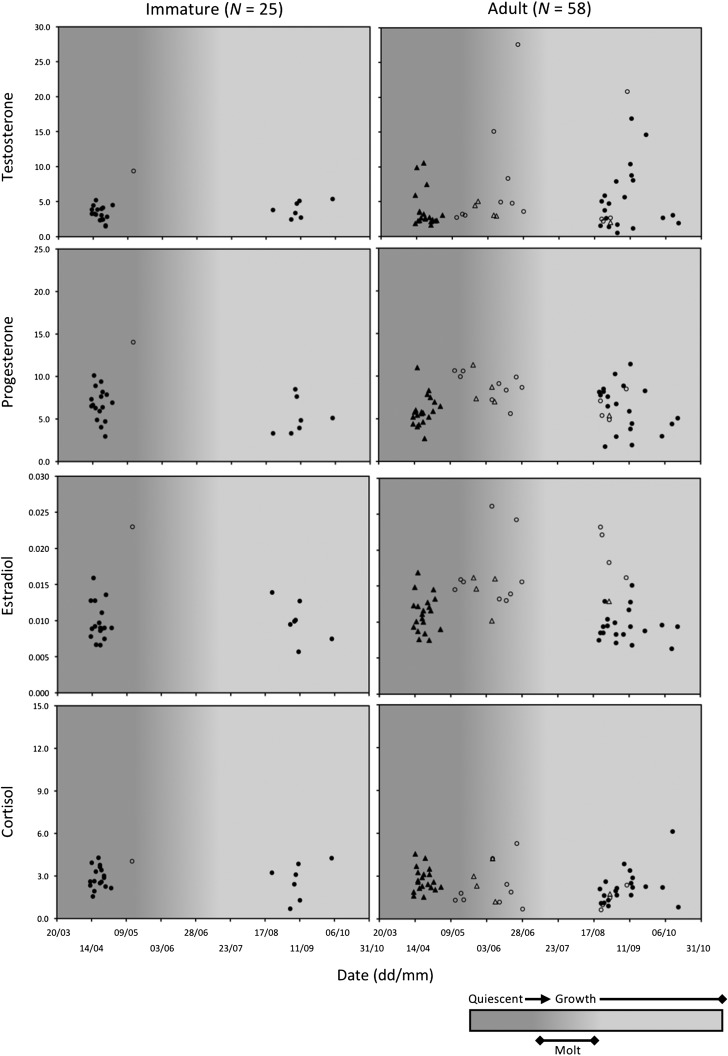
Hair hormone levels in 83 female brown bears in relation to time of year and phase of hair growth cycle (quiescent, molt, growth). Open circles and triangles represent bears captured in Alberta, Canada (*N* = 19). Closed circles and triangles represent bears captured or killed in Sweden (*N* = 64). Females < 3 years old were classified as immature whereas females ≥3 years old, which included solitary females (circles, *N* = 33) and all females with offspring (triangles, *N* = 25), were classified as adult.

Some patterns were evident when comparing observed hair hormone concentrations between age classes, and in relation to time of year (Figs 1 and 2). Within males, adult bears had higher testosterone values than immature bears (Fig. 1). Progesterone values in both age classes were higher in spring when hair growth was arrested (quiescent phase) than in fall when hair was growing (growth phase). The apparent seasonal difference in adult progesterone (and estradiol) levels may have also been caused by study area, given that ordinal day and study area were confounded for this age class. A seasonal pattern, higher values in spring than fall, was also apparent in the cortisol values for immature males, but not for adult males.

As with males, adult females had higher testosterone values than immature females (Fig. 2). It should be noted that hair testosterone concentrations in two adult females were greater than the highest value (18.1 pg/mg) recorded for adult males (Figs 1 and 2), although we were not comparing between sexes in this study. In addition, among adult females, testosterone values were greater in solitary bears than in bears accompanied by dependent offspring. Because differences in the hormone profiles of these two adult reproductive classes could potentially obscure differences between age classes, we had initially attempted to discriminate between three female age and reproductive classes, but invariably ended up with models that were too complicated for the data set, i.e. overfit models. Consequently, we abandoned this effort and limited our analyses to age classes. No clear patterns were evident based on the progesterone, estradiol and cortisol concentrations presented in Fig. 2, but the values for a single immature female sampled in Alberta were conspicuously high relative to values for immature females sampled in Sweden.

Among male bears, a candidate model based on a 3-year-old adult threshold was most supported (∆AIC_C_ = 0.00), although there was also support (∆AIC_C_ = 0.46) for a model based on a 5-year-old threshold (Table 3a). Nonetheless, measures of classification accuracy were greater when using the 3-year-old threshold (Table 3b), so this was the adult-age threshold that we used for subsequent analyses. Measures of classification accuracy showed little change when excluding the records of six 3-year-old bears in a re-analysis of the data (Table 3b). However, the most supported model changed from a ‘hormone + time’ model when using all data to a ‘hormone + contact method’ model when excluding the records of 3 years old (Table 3a).

As with male bears, the 3-year-old adult threshold model was also most supported for female bears, with no support (∆AIC_C_ > 2.00) for other adult-age threshold models (Table 4a). In a re-analysis of the data, with the records of six 3 years old excluded, the measures of performance accuracy changed little (Table 4b), but the most supported model changed from a ‘hormone + study area’ model to a ‘hormone with interactions’ model (Table 4a).

**Figure 3:**
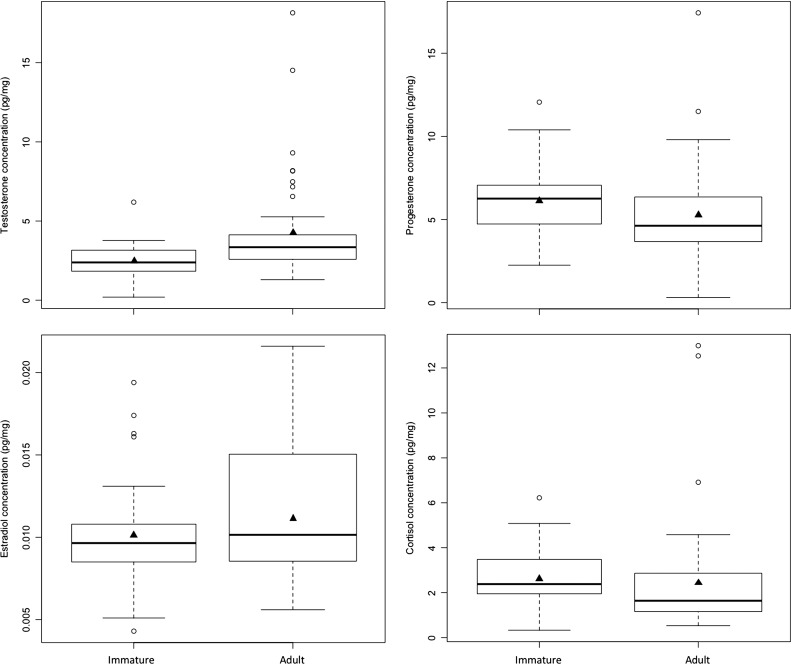
Comparison of hair hormone concentrations by age class in 86 male brown bears. Males < 3 years old were classified as immature (*N* = 42) whereas males ≥3 years old were classified as adult (*N* = 44). The box-and-whisker plots provide: (i) the median represented by a thick horizontal line; (ii) the interquartile range represented by the box; (iii) the minimum and maximum values, excluding outliers, represented by the lower and upper whiskers; and (iv) outliers being less than or greater than 1.5 times the lower and upper quartiles, represented by the open circles. The solid triangles represent the mean concentrations.

We found most support (∆AIC_C_ = 0.00) for a ‘hormone + time’ model (M4) to predict the age class of male brown bears (Table 5a). No other models were supported (∆AIC_C_ > 2.00). Model M4 included all four hormones, as well as interactions between progesterone and estradiol and between cortisol and time. Of these, the only parameters that were not statistically significant (*P* > 0.05) were the polynomial forms of estradiol (est^3^) and cortisol (cort^3^) (Table S3). Among significant parameters, testosterone levels were more likely to be higher in adults (odds ratio [OR] = 5.83), whereas progesterone and cortisol levels were more likely to be higher in immature bears (OR_Progesterone_ = 0.22, OR_Cortisol_ < 0.01) (Fig. 3 and Table S3). Further, the difference in cortisol levels between age classes was more pronounced during spring than fall (OR_Cortisol × Day_ = 6.56) (Fig. 1). All measures of classification accuracy were high for model M4, indicating a high rate of success in discriminating between immature and adult males (Table 5a and b, Fig. S3).

**Figure 4:**
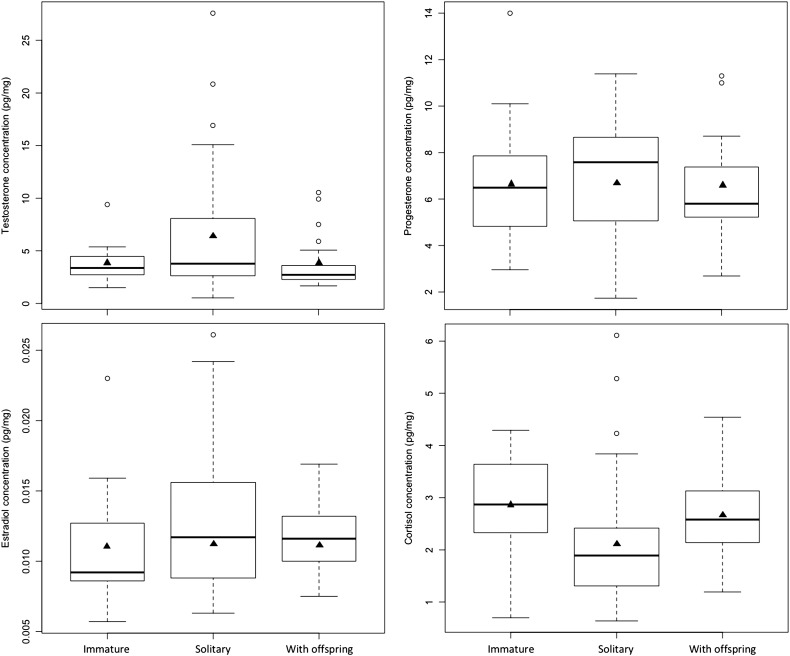
Comparison of hair hormone concentrations by age and reproductive class in 83 female brown bears. Females < 3 years old were classified as immature whereas females ≥3 years old, which included solitary females (*N* = 33) and all females with offspring (*N* = 25), were classified as adult. The box-and-whisker plots provide: (i) the median represented by a thick horizontal line; (ii) the interquartile range represented by the box; (iii) the minimum and maximum values, excluding outliers, represented by the lower and upper whiskers; and (iv) outliers being less than or greater than 1.5 times the lower and upper quartiles, represented by the open circles. The solid triangles represent the mean concentrations.

The age class of female brown bears was best predicted by a ‘hormone + study area’ model (F6) with no support (∆AIC_C_ > 2.00) for competing candidate models (Table 6a). Model F6 included testosterone and cortisol, as well as interactions between the two hormones and between testosterone and study area (Table S4). Among significant parameters, testosterone levels were more likely to be higher in adults (OR_Testosterone_ > 100), whereas cortisol levels were more likely to be higher in immature bears (OR_Cortisol_ = 0.30) (Fig. 4, Table S4). When comparing between adult reproductive classes, testosterone levels were higher and cortisol levels were lower in solitary females than in females with offspring (Fig. 4). The difference in testosterone levels between age classes appeared to be more pronounced in Sweden than in Alberta (OR_Testosterone × Study area_ > 100). However, this may have been artefactual, because only one immature female was sampled in Alberta, and the testosterone concentration for this individual (9.40 pg/mg) was higher than most of the concentrations recorded for adult females in Alberta (mean ± SE: 6.62 ± 1.69 pg/mg), with only 3 of 18 adult values exceeding this concentration. In contrast, the mean testosterone concentration in females sampled in Sweden was less for immature bears (3.48 ± 0.22 pg/mg) than for adults (4.59 ± 0.68 pg/mg). Measures of classification accuracy were not as high for model F6, or for female models in general (Table 6a and b, Fig. S4), when compared to the models for males shown in Table 5a and b. Overall, we had reasonable success in discriminating female age classes (mean AUC = 0.87), but we had considerably less success in correctly classifying immature females (sensitivity = 60%) than in correctly classifying adults (sensitivity = 85%).

## Discussion

The findings of this study suggest that measurement of steroid hormone concentration profiles in brown bear hair could be used to effectively determine age class, despite the significant constraints that we encountered with our data set. Because of the opportunistic manner by which we acquired hair samples, our data were highly unbalanced. Consequently, we were unable to completely separate the potential influences of the non-hormone factors—time, contact method and study area. We sidestepped this issue to some extent by avoiding the evaluation of candidate models using combinations of these confounding variables. The use of simpler models was also necessary given our small sample size. However, avoiding complicated models did not make the interpretation of models with hormone × non-hormone factor interactions any more certain. For example, in the testosterone × study area interaction in model F6 in Table 6a, we could not rule out the possibility that time and/or contact method also interacted with testosterone levels. It was fortuitous then that the individual hormones, and not their interactions with each other or with non-hormone factors, showed the strongest associations with male and female age class.

Another concern stemming from the opportunistic sampling was the variable time (6 months to 14 years) between the collection and laboratory analysis of samples. If hormone degradation had occurred at a relatively constant rate over time, hormone levels should have been lower in older than newer samples, when controlling for everything else. In our statistical analyses, we used year as a continuous variable to allow us to determine if there were any temporal trends over the 14 years that hair was collected. We reasoned that if hormone degradation occurred, we would find significant year × hormone interactions in the supported models (∆AIC_C_ ≤ 2.00) for male and female bears, but this did not occur. We also conducted four separate analyses in which the concentrations of the individual hormones were used as response variables, and sex-reproductive class and age were used as potential predictor variables (Tables S7–S10). With these analyses, we predicted that we would find a significant positive association between hormone concentration and year, if hormone degradation was significant. Again, however, year never appeared as a fixed effect, or as an interaction term, in any of the supported models. Thus, we have no evidence for hormone degradation influencing our findings. Although we are unaware of any studies that have evaluated the effect of storage time on reproductive hormone concentrations in hair, Macbeth *et al.* (2010) previously demonstrated that cortisol remains stable in brown bear hair for at least 17 months. Cortisol has also been extracted from museum samples of hair collected from polar bears that were killed 85–120 years ago, and concentrations in these samples were found to be generally higher than the range of values measured in hair collected from polar bears from the same area in recent years (Bechshøft *et al.*, 2012).

The capability to correctly classify the age class of brown bears based on hormone concentrations in hair samples collected non-invasively is a significant finding with important implications for brown bear conservation. Knowledge of age structure is essential to accurately assess population demographics (Doak and Cutler, 2014) and the success of recovery efforts (Woodruff *et al.*, 2016). It is also essential for determining sustainable harvest rates for exploited species, including brown bears (Gosselin *et al.*, 2015; McLellan *et al.*, 2017). Further, for populations-at-risk, estimates of juvenile survival are often a strong indicator of their performance and viability (Woodruff *et al.*, 2016). Assigning age to an individual animal usually involves capture and handling but this can be expensive, dangerous to field personnel and harmful to the animal (Arnemo *et al.*, 2006; Cattet *et al.*, 2008). With non-invasive sampling, age determination may be possible for some, but not all, species by camera trapping (Vaz Pinto *et al.*, 2016) or faecal morphometry (Woodruff *et al.*, 2016). However, animals cannot be reliably aged through the molecular analysis (e.g. telomere length) of hair or faecal samples, although the pursuit of molecular age biomarkers continues to be an active field of research (Jarman *et al.*, 2015). Future research should address the possibility to use hair hormone concentrations to differentiate age classes at a finer scale to better meet management needs, e.g. cubs-of-the year vs other immature bears vs one or more adult classes.

We found more support for adulthood beginning at 3 years, than at 4 or 5 years, in both sexes in this study. We selected 3 years as the lowest adult-age threshold to evaluate, because databases from both projects contained records of female bears at 4 years accompanied by cubs-of-the-year, which means they would have bred at 3 years, and because male bears in Sweden have been determined by DNA profiling (microsatellite analysis) to breed as early as 3 years (Zedrosser *et al.*, 2007). We recognized, however, that the median age threshold could also be higher, because of variation among individual bears in the timing of sexual maturation (Schwartz *et al.*, 2003). In this regard, several studies of brown bears in the study areas from where we obtained samples have also used 4 or 5 years as the adult-age threshold (Zedrosser *et al.*, 2013; Gosselin *et al.*, 2015; Sorensen *et al.*, 2015; Kite *et al.*, 2016).

The primary process by which hormones accumulate in hair raises a question as to whether the hormone profile in a brown bear hair sample reflects the current endocrine state of the animal or its endocrine state at some previous point in time. Although not fully investigated, hormones are presumed to sequester in growing hair primarily through passive diffusion from the systemic blood circulation to the follicular cells that produce the hair shaft (Sharpley *et al.*, 2011; Russell *et al.*, 2012). Local skin production of hormones may also contribute, but to a lesser degree, through local blood flow (Zouboulis, 2009; Inoue *et al.*, 2012; Slominski *et al.*, 2013), and possibly in association with sebum and sweat penetrating the hair shaft above the skin (Russell *et al.*, 2014). Nonetheless, irrespective of the relative contributions from the systemic blood circulation and local skin production, hormones should be sequestered in brown bear hair when it is growing, not when it is quiescent. As depicted in Figs 1 and 2, the hair cycle in brown bears can be broken down into a growth phase from June to October, and a quiescent phase from November to May. Molt occurs during May and June, such that hair samples collected during this time are likely to be a mixture of quiescent and growing hairs. However, the timing of hair growth in brown bears shows considerable plasticity that depends on the quantity and quality of the diet (Jacoby *et al.*, 1999), which is why the hair cycle phases were illustrated as gradients in Figs 1 and 2. In this study, four of twelve 3-year-old bears, two males and two females, were sampled in May and June coincident with the molt and early growth, whereas the other eight 3 years old were sampled during August and September, further into the growth phase. Because the endocrine profiles of the four samples collected during May and June could have reflected the endocrine state of the bears during the previous fall, when they were 2 years old, we excluded the records of all 3-year-old bears and re-analysed the data to see if classification accuracy improved. It did not, but this was not surprising, given that only four 3 years old were sampled during the quiescent and early growth phases. Still, we cannot be sure that classification accuracy would have improved if more 3-year-old bears had been sampled during the quiescent phase. On this point, Cattet *et al.* (2017) measured marked changes (2- to 3-fold increases and decreases) in the hair concentrations of reproductive hormones in captive brown bears that were repeatedly sampled (two to three times) during the same quiescent phase. This points to the need for more research to better understand how and when hormones accumulate in hair.

Male brown bears were separated into immature and adult classes with a high degree of accuracy (mean AUC > 0.95) based on the concomitant concentrations of all four hormones in their hair. Our interpretation of these findings in the broader context of brown bear reproductive endocrinology is somewhat limited, because of the relative lack of published information on this topic, not just in males, but females as well. We are unaware of any studies of brown bears that have compared reproductive hormone levels by male age class in any type of biological sample. In polar bears, the closest relative to brown bears, serum testosterone concentrations are positively correlated with age during the breeding season (April–May), but do not differ during October, a time when bears are not reproductively active (Palmer *et al.*, 1988). In this study of free-ranging brown bears, we found that hair testosterone levels were generally higher in adult males (4.32 ± 0.49 pg/mg, *N* = 44) than in immature males (2.54 ± 0.16 pg/mg, *N* = 42) from April to October, without any evidence of a temporal pattern (Fig. 1). In contrast, Cattet *et al.* (2017) found the hair testosterone concentrations in two captive adult male brown bears was highest (≥7 pg/mg) from April to June, which coincided with breeding, but remained at low levels (<3 pg/mg) from August to February. In this same study, progesterone (range: 2.28–5.81 pg/mg, *N* = 10) and estradiol concentrations (0.005–0.019 pg/mg; *N* = 10) were also measured, and the observed values were similar to what we recorded for free-ranging adult males (Fig. 1). In contrast, however, hair cortisol levels were lower in captive adult males (1.08 ± 0.12 pg/mg, *N* = 10) than in free-ranging adult males (2.52 ± 0.39 pg/mg, *N* = 44).

**Figure 5:**
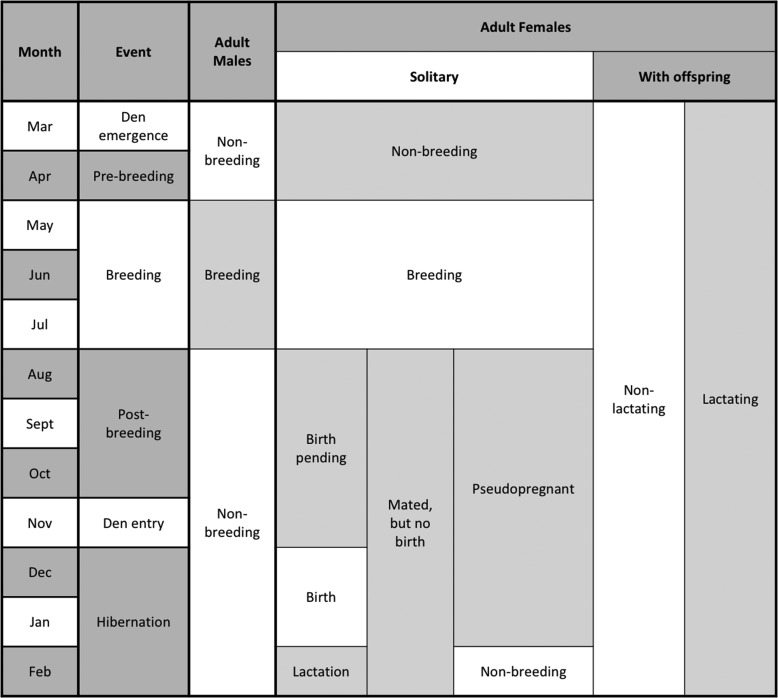
Comparison of potential reproductive states between adult males and adult females during different months of the year. This characterization of the annual reproductive cycle of brown bears is based on a review of reproductive seasonality in bears by Spady *et al.* (2007).

Our ability to correctly discriminate between age classes was less accurate for females than for males. Although we had good success in correctly classifying adult females (sensitivity = 85%), our success in correctly classifying immature females was poor (sensitivity = 60%). We suggest that the greater complexity of the reproductive cycle in females versus males may have reduced our success in classifying females simply on the basis of age class (Fig. 5). Hormonal changes in adult males are restricted to the time prior to breeding, from April to mid-May when the testicles are growing and differentiating in preparation for spermatogenesis, and during breeding in late May and June (Tsubota and Kanagawa, 1989; Spady *et al.*, 2007). Consequently, adult males can only be identified as breeding or non-breeding on the basis of their hair hormone profile, as previously shown in captive bears (Cattet *et al.*, 2017). In contrast, adult females can exist in multiple reproductive states that are spread throughout much of the year (Fig. 5). Females with offspring can be either lactating or not in any month of the active season (March–November), but solitary females may be in one of four possible states—breeding, pregnant, non-pregnant or pseudopregnant—during this time. And presumably, these different reproductive states are characterized by different hair hormone profiles. In captive brown bears, the hair reproductive hormone profiles of adult females differed between non-breeding, breeding, and pregnant states (Cattet *et al.*, 2017). Although we did not include reproductive state in the analyses of this study, it appears from Figs 2 and 4 that the hair hormone profiles of solitary adult females and females with offspring differed, and that these differences may have blurred the distinction between age classes. This could be verified through further study with a larger sample of adult females of known reproductive state. In addition, it would be invaluable to track the hair hormone profiles of immature females, that were previously captured and are of known age, through multi-year non-invasive genetic sampling to identify specific changes in hormone levels that are associated with their transition to adulthood.

Female age class was most closely associated with concurrent hair concentrations of testosterone and cortisol. Surprisingly, neither progesterone nor estradiol occurred in the most supported model, although it these two hormones that have been evaluated most often in studies of female brown bears (Tsubota *et al.*, 1987, 1992; Wasser *et al.*, 2004; Dehnhard *et al.*, 2006). Broadly speaking, studies of females across many species, including humans, have focused on progesterone and estradiol, whereas testosterone is usually associated with studies of males (Arnon *et al.*, 2016). Nevertheless, we have shown in a previous study with captive brown bears that hair testosterone levels in adult females, whether breeding or not, change markedly throughout the year (Cattet *et al.*, 2017). Further, as found in this study of free-ranging bears, the hair testosterone levels of captive adult females may exceed the levels measured in adult males. High concentrations of hair testosterone in females, relative to levels in males, have also been recorded for wolves (*Canis lupus*; Bryan *et al.*, 2014), Canada lynx (*Lynx canadensis*; Terwissen *et al.*, 2014), and ring-tailed lemurs (*Lemur catta*; Tennenhouse *et al.*, 2016). Although the significance of ‘high testosterone levels’ in female brown bears has not been explored, attention has been directed to the potential adaptive consequences of maternal testosterone levels on offspring behaviour (Dloniak *et al.*, 2006) and sex ratios (Grant and Chamley, 2010; Edwards *et al.*, 2016) in other mammals.

The direct application of our preliminary findings to non-invasive genetic studies may be prevented at present by the large quantity of hair required for the hormone analyses. We required ~125 mg of guard hair (~125–250 individual guard hairs) per sample to reliably measure the four hormones evaluated in this study. Although this amount can be collected easily from a captured or killed bear, it far exceeds the amount (≤30 mg) that is typically snagged by a single barb of barbed wire. Nevertheless, it may be possible to resolve this problem by combining several approaches. The first would be to maximize the amount of hair collected per animal by using multiple strands of barbed wire and/or using wire with closely spaced (5 cm) barbs. The second would be to determine the ease and reliability of analysing mixed (guard and undercoat) hair samples instead of selecting and analysing guard hairs only. Finally, the application of mass spectrometry methods, which are considered to be the ‘gold standard’ for hair analysis (Gow *et al.*, 2010), instead of enzyme-linked immunosorbent assays (ELISA), as were used for this study, would enable the concurrent measurement of multiple hormones in a considerably smaller amount of hair (≥20 mg) than was used in this study (Gao *et al.*, 2013). Alternatively, it may be possible to reduce the amount of hair required for ELISA through modifications to the extraction protocol, e.g. increase the solvent–sample ratio, longer incubation times.

In conclusion, we have demonstrated that the measurement of hormone profiles in the hair of brown bears has potential to be applied to augment DNA-based capture-mark-recapture studies by enabling accurate assignment of age class to male bears. However, our capability to discriminate between immature and adult females was less reliable, which points toward the need for additional research based on a larger sample size with balanced representation among female age and reproductive classes. Beyond enhancing DNA-based capture-mark-recapture studies, the capability to discriminate among adult female reproductive states would also contribute to understanding social, demographic and ecological processes in brown bears. Given the small and unbalanced sample used in this study, our findings should be viewed as preliminary, but they should also provide a basis for more comprehensive future studies.

## Supplementary Material

Supplementary Data
