# Supplementary material for: Can concentrations of steroid hormones in brown bear hair reveal age class?
Source: Conserv Physiol. 2018 Jan 29;6(1):coy001. doi: 10.1093/conphys/coy001 (PMC5788069; doi:10.1093/conphys/coy001)
Supplement: Supplementary Data [file conphys-2017-041_supp_rev1.docx]

Table S1. Classification of 86 records of hair collected from male brown bears in Alberta, Canada, and Sweden into categories by age class, study area, contact method, and season.

| Age class^a^ | Study area | Contact method | Season^b^ | Number of records |
| --- | --- | --- | --- | --- |
| Immature | Alberta | Helicopter | Pre-breeding | 0 |
|  |  |  | Breeding | 1 |
|  |  |  | Post-breeding | 0 |
|  |  | Culvert trap | Pre-breeding | 0 |
|  |  |  | Breeding | 1 |
|  |  |  | Post-breeding | 1 |
|  |  | Killed by gunshot | Pre-breeding | 0 |
|  |  |  | Breeding | 0 |
|  |  |  | Post-breeding | 0 |
|  | Sweden | Helicopter | Pre-breeding | 24 |
|  |  |  | Breeding | 2 |
|  |  |  | Post-breeding | 0 |
|  |  | Culvert trap | Pre-breeding | 0 |
|  |  |  | Breeding | 0 |
|  |  |  | Post-breeding | 0 |
|  |  | Killed by gunshot | Pre-breeding | 0 |
|  |  |  | Breeding | 0 |
|  |  |  | Post-breeding | 13 |
| Adult | Alberta | Helicopter | Pre-breeding | 0 |
|  |  |  | Breeding | 2 |
|  |  |  | Post-breeding | 1 |
|  |  | Culvert trap | Pre-breeding | 0 |
|  |  |  | Breeding | 10 |
|  |  |  | Post-breeding | 1 |
|  |  | Killed by gunshot | Pre-breeding | 0 |
|  |  |  | Breeding | 0 |
|  |  |  | Post-breeding | 0 |
|  | Sweden | Helicopter | Pre-breeding | 0 |
|  |  |  | Breeding | 0 |
|  |  |  | Post-breeding | 0 |
|  |  | Culvert trap | Pre-breeding | 0 |
|  |  |  | Breeding | 0 |
|  |  |  | Post-breeding | 0 |
|  |  | Killed by gunshot | Pre-breeding | 0 |
|  |  |  | Breeding | 0 |
|  |  |  | Post-breeding | 30 |

^a^ Bears <3-years old were classified as immature whereas bears ≥3-years old were classified as adult.

^b^ Date ranges for seasons were den emergence to April 30^th^ for pre-breeding, May 1^st^ to July 15^th^ for breeding, and July 16^th^ to den entry for post-breeding.

Table S2. Classification of 83 records of hair collected from female brown bears in Alberta, Canada, and Sweden into categories by age and reproductive class, study area, contact method, and season.

| Age and reproductive class^a^ | Study area | Contact method | Season^b^ | Number of records |
| --- | --- | --- | --- | --- |
| Immature | Alberta | Helicopter | Pre-breeding | 0 |
|  |  |  | Breeding | 0 |
|  |  |  | Post-breeding | 0 |
|  |  | Culvert trap | Pre-breeding | 0 |
|  |  |  | Breeding | 1 |
|  |  |  | Post-breeding | 0 |
|  |  | Killed by gunshot | Pre-breeding | 0 |
|  |  |  | Breeding | 0 |
|  |  |  | Post-breeding | 0 |
|  | Sweden | Helicopter | Pre-breeding | 17 |
|  |  |  | Breeding | 0 |
|  |  |  | Post-breeding | 0 |
|  |  | Culvert trap | Pre-breeding | 0 |
|  |  |  | Breeding | 0 |
|  |  |  | Post-breeding | 0 |
|  |  | Killed by gunshot | Pre-breeding | 0 |
|  |  |  | Breeding | 0 |
|  |  |  | Post-breeding | 7 |
| Solitary adult | Alberta | Helicopter | Pre-breeding | 0 |
|  |  |  | Breeding | 4 |
|  |  |  | Post-breeding | 3 |
|  |  | Culvert trap | Pre-breeding | 0 |
|  |  |  | Breeding | 5 |
|  |  |  | Post-breeding | 1 |
|  |  | Killed by gunshot | Pre-breeding | 0 |
|  |  |  | Breeding | 0 |
|  |  |  | Post-breeding | 0 |
|  | Sweden | Helicopter | Pre-breeding | 0 |
|  |  |  | Breeding | 0 |
|  |  |  | Post-breeding | 0 |
|  |  | Culvert trap | Pre-breeding | 0 |
|  |  |  | Breeding | 0 |
|  |  |  | Post-breeding | 0 |
|  |  | Killed by gunshot | Pre-breeding | 0 |
|  |  |  | Breeding | 0 |
|  |  |  | Post-breeding | 20 |
| With offspring | Alberta | Helicopter | Pre-breeding | 0 |
|  |  |  | Breeding | 3 |
|  |  |  | Post-breeding | 1 |
|  |  | Culvert trap | Pre-breeding | 0 |
|  |  |  | Breeding | 1 |
|  |  |  | Post-breeding | 0 |
|  |  | Killed by gunshot | Pre-breeding | 0 |
|  |  |  | Breeding | 0 |
|  |  |  | Post-breeding | 0 |
|  | Sweden | Helicopter | Pre-breeding | 19 |
|  |  |  | Breeding | 1 |
|  |  |  | Post-breeding | 0 |
|  |  | Culvert trap | Pre-breeding | 0 |
|  |  |  | Breeding | 0 |
|  |  |  | Post-breeding | 0 |
|  |  | Killed by gunshot | Pre-breeding | 0 |
|  |  |  | Breeding | 0 |
|  |  |  | Post-breeding | 0 |

^a^ Bears <3-years old were classified as immature whereas bears ≥3-years old, including all females with offspring, were classified as adult.

^b^ Date ranges for seasons were den emergence to April 30^th^ for pre-breeding, May 1^st^ to July 15^th^ for breeding, and July 16^th^ to den entry for post-breeding.

Table S3. Standardized^a^ coefficients (β), standard errors (SE), and odds ratios (OR) for the estimable parameters^b^ used in the most supported predictive model (M4) for male brown bears (*N* = 86) shown in Table 5a. Bold typeface denotes β- and OR-values that were statistically significant at *P* ≤ 0.05.

| Estimable parameter | β | SE | OR |
| --- | --- | --- | --- |
| intercept | **-2.02** | **0.91** | **0.13** |
| test | **4.07** | **1.32** | **5.83** |
| prog | **-1.51** | **0.70** | **0.22** |
| est^3^ | 0.13 | 0.12 | 0.00 |
| cort | **-7.86** | **2.61** | **<0.01** |
| cort^3^ | 4.28 | 2.26 | 7.21 |
| d | **3.21** | **1.10** | **2.48** |
| prog × est^3^ | **0.50** | **0.21** | **0.00** |
| cort × d | **4.18** | **1.56** | **6.56** |

^a^ We standardized all continuous predictor variables by subtracting the mean from the observed values and dividing by the standard deviation. This was done to reduce multicollinearity and the associated problems that are caused by two-way interactions when calculating β-coefficients.

^b^ Variables are testosterone (test), progesterone (prog), estradiol (est), cortisol (cort), and ordinal day (d).

Table S4. Standardized^a^ coefficients (β), standard errors (SE), and odds ratios (OR) for the estimable parameters^b^ used in the most supported predictive model (F6) for female brown bears (*N* = 83) shown in Table 6a. Bold typeface denotes β- and OR-values that were statistically significant at *P* ≤ 0.05.

| Estimable parameter | β | SE | OR |
| --- | --- | --- | --- |
| intercept | 0.29 | 0.55 | 0.00 |
| test | **-8.67** | **3.87** | **<0.01** |
| test^2^ | **12.20** | **4.38** | **>100.00** |
| cort | **-1.21** | **0.59** | **0.30** |
| cort^3^ | -0.32 | 0.39 | 0.73 |
| test × cort^3^ | -1.42 | 0.75 | 0.24 |
| test × sa (Sweden) | **14.98** | **6.18** | **>100.00** |

^a^ We standardized all continuous predictor variables by subtracting the mean from the observed values and dividing by the standard deviation. This was done to reduce multicollinearity and the associated problems that are caused by two-way interactions when calculating β-coefficients.

^b^ Variables are testosterone (test), cortisol (cort), and study area (sa).

Table S5. Classification table for the most supported model, M1, for male brown bears from Table 5a.

|  |  | OBSERVED | |  |
| --- | --- | --- | --- | --- |
|  |  | Immature male | Adult male |  |
| PREDICTED | Immature male | 35 | 3 | 38 |
|  | Adult male | 7 | 41 | 48 |
|  | Total | 42 | 44 | 86 |
|  | Correct | 35 | 41 | 76 |
|  | Correct (%) | 83.3 | 93.2 | 88.4 |

Table S6. Classification table for the most supported model, F1, for female brown bears from Table 4a.

|  |  | OBSERVED | |  |
| --- | --- | --- | --- | --- |
|  |  | Immature female | Adult female |  |
| PREDICTED | Immature female | 15 | 9 | 24 |
|  | Adult female | 10 | 49 | 59 |
|  | Total | 25 | 58 | 83 |
|  | Correct | 15 | 49 | 64 |
|  | Correct (%) | 60.0 | 84.5 | 77.1 |

Table S7. Comparison of candidate models by Aikaike Information Criteria (AIC) model selection to predict the hair testosterone concentration for 169 free-ranging brown bears.

| Model | *K* | AIC_C_ | ∆AIC_C_ | *w_i_* |
| --- | --- | --- | --- | --- |
| 6. Hormone + sex-repro + age + day + contact method | 11 | 684.05 | 0.00 | 0.81 |
| 3. Hormone + sex-repro + age | 8 | 688.86 | 4.81 | 0.07 |
| 4. Hormone + sex-repro + age + day | 9 | 688.93 | 4.88 | 0.07 |
| 5. Hormone + sex-repro + age + day + year | 10 | 690.81 | 6.76 | 0.03 |
| 7. Hormone + sex-repro + age + day + country | 10 | 691.15 | 7.10 | 0.02 |
| 2. Hormone only | 5 | 712.08 | 28.03 | 0.00 |
| 1. Null (intercept only) | 2 | 763.19 | 79.15 | 0.00 |

Table S8. Comparison of candidate models by Aikaike Information Criteria (AIC) model selection to predict the hair progesterone concentration for 169 free-ranging brown bears.

| Model | *K* | AIC_C_ | ∆AIC_C_ | *w_i_* |
| --- | --- | --- | --- | --- |
| 7. Hormone + sex-repro + age + day + country | 10 | 748.55 | 0.00 | 0.81 |
| 6. Hormone + sex-repro + age + day + contact method | 11 | 752.75 | 4.20 | 0.10 |
| 5. Hormone + sex-repro + age + day + year | 10 | 753.87 | 5.32 | 0.06 |
| 4. Hormone + sex-repro + age + day | 9 | 755.08 | 6.53 | 0.03 |
| 3. Hormone + sex-repro + age | 8 | 772.36 | 23.81 | 0.00 |
| 2. Hormone only | 5 | 778.60 | 30.05 | 0.00 |
| 1. Null (intercept only) | 2 | 798.98 | 50.43 | 0.00 |

Table S9. Comparison of candidate models by Aikaike Information Criteria (AIC) model selection to predict the hair estradiol concentration for 169 free-ranging brown bears.

| Model | *K* | AIC_C_ | ∆AIC_C_ | *w_i_* |
| --- | --- | --- | --- | --- |
| 7. Hormone + sex-repro + age + day + country | 10 | -1513.18 | 0.00 | 1.00 |
| 6. Hormone + sex-repro + age + day + contact method | 11 | -1498.33 | 14.85 | 0.00 |
| 5. Hormone + sex-repro + age + day + year | 10 | -1485.15 | 28.03 | 0.00 |
| 2. Hormone only | 5 | -1456.70 | 56.49 | 0.00 |
| 7. Hormone + sex-repro + age | 8 | -1452.42 | 60.77 | 0.00 |
| 2. Hormone + sex-repro + age + day | 9 | -1450.19 | 63.00 | 0.00 |
| 1. Null (intercept only) | 2 | -1415.36 | 97.82 | 0.00 |

Table S10. Comparison of candidate models by Aikaike Information Criteria (AIC) model selection to predict the hair cortisol concentration for 169 free-ranging brown bears.

| Model | *K* | AIC_C_ | ∆AIC_C_ | *w_i_* |
| --- | --- | --- | --- | --- |
| 6. Hormone + sex-repro + age + day + contact method | 11 | 536.68 | 0.00 | 0.38 |
| 7. Hormone + sex-repro + age + day + country | 10 | 537.57 | 0.89 | 0.24 |
| 4. Hormone + sex-repro + age + day | 9 | 538.35 | 1.67 | 0.16 |
| 5. Hormone + sex-repro + age + day + year | 10 | 538.87 | 2.19 | 0.13 |
| 2. Hormone only | 5 | 540.27 | 3.59 | 0.06 |
| 3. Hormone + sex-repro + age | 8 | 541.67 | 4.99 | 0.03 |
| 1. Null (intercept only) | 2 | 559.99 | 23.31 | 0.00 |


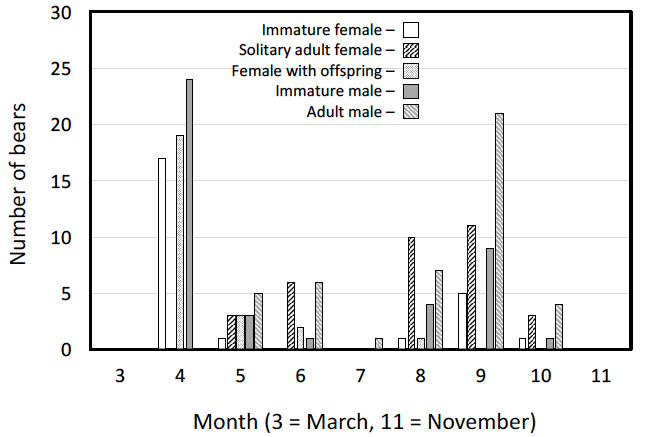


Figure S1. Number of free-ranging brown bears sampled by sex, age and reproductive class, and the distribution of sampling events by month.


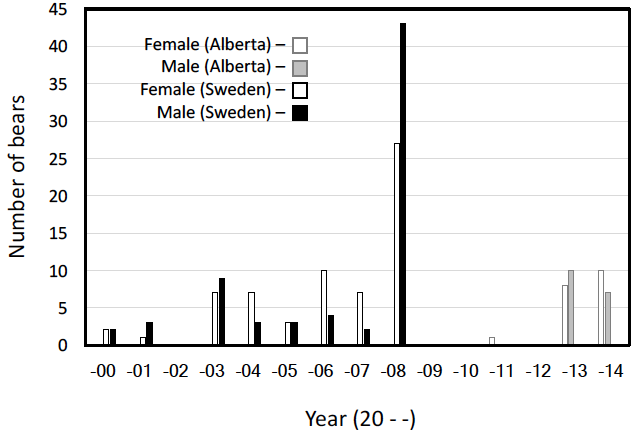


Figure S2. Number of free-ranging brown bears sampled by sex and study area, and the distribution of sampling events by year.


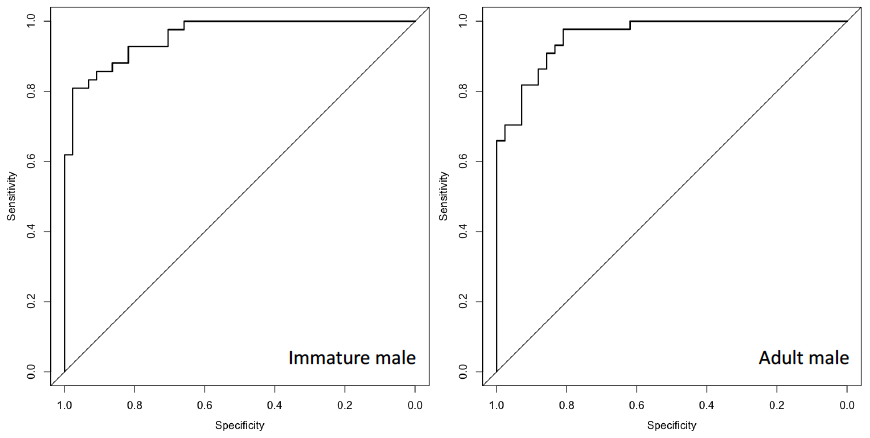


Figure S3. Receiver Operating Characteristic (ROC) curves for immature (*N* = 42) and adult males (*N* = 44) based on the top predictive model, M4, for male brown bears from Tables 5a and 5b.


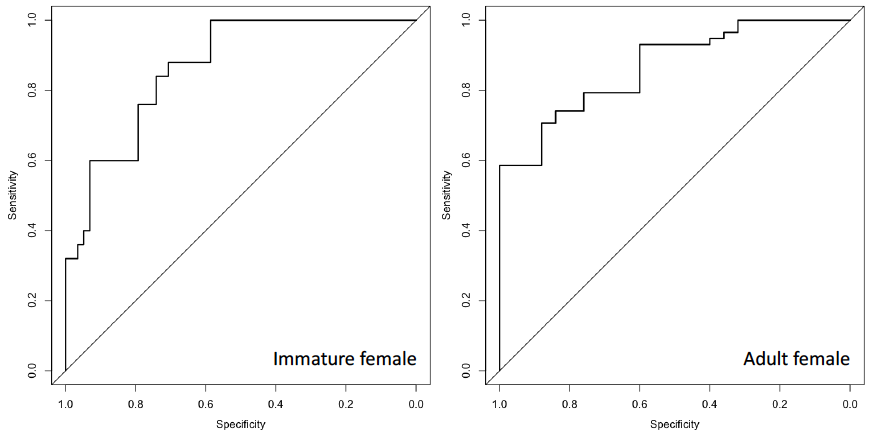


Figure S4. Receiver Operating Characteristic (ROC) curves for immature (*N* = 25) and adult females (*N* = 58) based on the top predictive model, F6, for female brown bears from Tables 6a and 6b.
